## [Peer Review File · Communications Biology]

Reviewers' comments:

Reviewer #1 (Remarks to the Author):

In this manuscript, Khilji and colleagues investigate the role of retinoid X receptor signaling during the early stages of skeletal myogenic differentiation. Following the initial identification of bexarotene as an agent promoting myogenic differentiation through regulation of MyoD and other genes (Hamed M et al Nucleic Acid Research, 2017), the authors extended their studies to determine whether RXR signaling regulates Myogenin genomic binding.

Overall, this manuscript is well written and the results are reported in a very clear way.

The main conceptual limitation is that bexarotene treatment increases the protein levels of Myogenin (which, based on the previous manuscript from the same group, is MyoD-dependent). For this reason, it is not clear whether the additional Myogenin binding sites detected upon 24h bexarotene treatment (Figure 1D) represent novel sites not previously available or they are detected due to a more efficient chromatin immunoprecipitation. In fact, the number of Myogenin peaks detected in each experiment is $\sim 1/4$ of the number of sites reported in the previous paper by the same group (Figure 5A of Hamed M et al) and the results observed in 24h bex-treated cells are similar to the changes in Myogenin expression and genomic binding in myogenic cells over a longer period of differentiation. Because the majority of the manuscript relies on the differences between these two types of Myogenin binding sites, it is important to clarify what promotes binding of Myogenin. One possibility is determine whether RXR primes within a short time the sites that will be later occupied by Myogenin by measuring, for example, chromatin accessibility followed by footprinting analysis. A more complex scenario would be that MyoD promotes Myogenin binding.

Minor comment

The statistical tool used in some of the analyses is not fully reported. Authors could add the details to the Figure legends or a section in the methods.

Reviewer #2 (Remarks to the Author):

In this work authors investigated the molecular mechanisms at the basis of the Retinoid X Receptor (RXR) signalling activation in muscle differentiation. In the first part of the work they performed the ChIP-seq of Myogenin in cells treated or not with the RXR-agonist bexarotene finding differences in MyoG binding profiles. In particular, upon RXR activation MyoG redistributes on chromatin and preferentially binds active and poised enhancers. Then, they analysed the P300 binding and the epigenetic signature associated at new Bex-dependent sites of binding. They found a P300 redistribution on promoters and an RXR dependent increase of H3K9ac and H4K8ac at enhancers occupied by both P300 and Myogenin. The authors speculate that these mechanisms could be important for muscle regeneration.

The study of epigenetic mechanisms underlying muscle differentiation is important to finely dissect the myogenesis dynamics and to modulate epigenetic functions to support muscle regeneration. Thus the field is of interest for the entire scientific community. However, I found that the main conclusions are not clearly supported by presented data.

I recommend a major revision prior the publication in Communication Biology.

I found the redistribution of Myogenin on the chromatin and the change in the consensus very interesting, but the major question regards the molecular mechanism. Does the Bex treatment

accelerate the muscle differentiation or is it forcing some molecular mechanisms that normally are repressed?

Supplementary Figure 3 suggests that RXR activation determines an acceleration of myogenesis, thus authors should also analyse the MT4 time point in C2C12 and to knock down the RXR receptor, to see if there are alternative compensatory mechanisms in the localization of MyoG on different targets.

Major comments:

- 1) Results section: Figure 2. The authors found a different consensus binding for MyoG in control of Bex-treated cells. This result should be confirmed with in vitro data. One possibility is to transfect constructs containing the 2 different consensus and to perform a ChIP of Myogenin with or without Bex and RXR depletion.
- 2) Results section: Figure S3. From the analysis of publicly available Myogenin ChIP-seq, the authors found 45% of consensus for Myogenin in C2C12 differentiated 24h while in the MyoG ChIP presented in the work they found 21%. Why they have only 50% of published binding sites? Is it possible that the quality of differentiation is poor? Maybe some quality controls on differentiation could help in the evaluation.
- 3) Results section: Figure 2 and S3. The authors should try to perform the Bex-treatment on primary myoblasts to prove the reliability of the treatment and the translational potential.
- 4) Results section: Figure S3. The authors stated: "In addition, it is only in primary myoblasts differentiated for 24 hours or C2C12 myoblasts for 7 days, where we discerned two additional consensus flanking nucleotides, mirrored by myoblasts differentiated with bexarotene for 24 hours (Fig. 2a, Supplementary Fig. 3a)". However, in primary myoblasts I can see only one additional consensus nucleotide.
- 5) Results section: Figure 2. The authors stated: "Moreover, myogenin loci associated with single Ebox generally appeared as sharper and narrower peaks compared to a broader compilation of read signals at loci containing double E-box, shown by Integrated Genome Viewer36 (IGV) snapshots of representative myogenin ChIP-seq tracks (Fig. 2f, g)". To reinforce this statement they should shown a zoom in of the region containing the peak.
- 6) Results section: Figure 4a. The authors stated: "Taken together, rexinoids promote differential recruitment of p300 to promoters and enhancers in that p300 may mediate RXR signaling through distinct functional modes". However, I could see an increase of the recruitment at promoters (not enhancers).
- 7) Results section: Figure 5c. P300 deposition seems to be less upon Bex treatment. Western blots with total P300 and histone marks levels can elucidate if bex-treatment increase the total amount of protein/histone or determine their redistribution on the genome.
- 8) Results section: Figure 5c. All histone marks increase upon Bex treatment. Since the ChIP-seq are not quantitative I recommend to look another histone modification (H3K9me3 or another repressive modification) at least to show on the same track a different trend. Alternatively quantitative ChIP are recommended (Orlando DA, Chen MW, Brown VE, Solanki S, Choi YJ, Olson ER, Fritz CC, Bradner JE, and Guenther MG. Quantitative ChIP-Seq normalization reveals global modulation of the epigenome. Cell Rep. 2014;9(3):1163-70).
- 9) Results section: Figure 6d. The enhancer should be clearly indicated. Authors should analyse a well-described enhancer (not putative) to reinforce their speculations.
- 10) A model to summarize all results can help the reader.

Minor comments:

- 1) References are not correctly formatted. Reference number 28 at page 5 line 6 "28. Boehm, M. F. et al. Synthesis and Structure-Activity Relationships of Novel Retinoid X Receptor-Selective Retinoids. Journal of Medicinal Chemistry 37, 2930–2941 (1994)" seems to incorrectly substitute the number 30 "30. AlSudais, H. et al. Retinoid X Receptor-selective Signaling in the Regulation of Akt/Protein Kinase

B Isoform-specific Expression. Journal of Biological Chemistry 291, 3090–3099 (2016).”

2) Results section: Figure 1a and b should be placed in supplementary, because the increase of Myogenin upon Bex treatment was already shown in the Figure 2 of a previous work: “30. AlSudais, H. et al. Retinoid X Receptor-selective Signaling in the Regulation of Akt/Protein Kinase B Isoform-specific Expression. Journal of Biological Chemistry 291, 3090–3099 (2016)”.

3) Results section: In Figure 1 a track of Myogenin could help the reader to evaluate the ChIP-seq.

4) Results section: Figure 4a. The legend of the figure is necessary to evaluate the graph.

5) Figure 6d: From the color of the map I supposed that the enhancer they are analyzing was classified by Chrom-HMM as poised enhancer. This should be clear in the figure, maybe authors could add a color legend.

QIAO LI MD, PhD

613 562 5800 ext. 8491
Qiao.Li@uOttawa.ca

March 9, 2020

We thank the reviewers sincerely for their constructive critique and valuable suggestions, and are encouraged by the positive comments, such as *“this manuscript is well written and the results are reported in a very clear way”*; *“The study of epigenetic mechanisms underlying muscle differentiation is important to finely dissect the myogenesis dynamics and to modulate epigenetic functions to support muscle regeneration. Thus the field is of interest for the entire scientific community”*; *“the redistribution of Myogenin on the chromatin and the change in the consensus very interesting”*. We have now provided additional data as requested and attempted to address all the concerns raised by the reviewers. The incorporation of their suggestions has substantially improved our manuscript and we hope you will find that the manuscript has now met the standard for publication. Specific response are as follows:

Reviewer #1:

The reviewer commented that *“it is not clear whether the additional Myogenin binding sites detected upon 24h bexarotene treatment (Figure 1D) represent novel sites not previously available or they are detected due to a more efficient chromatin immunoprecipitation”*. The same stringent ChIP procedure was applied for both treated and untreated conditions, and the program used to identify unique peak positions compares the coordinates of peaks from each condition. Therefore, peaks unique to bexarotene likely *“represent novel sites not previously available”*, and not due to a more efficient ChIP. With respect to the number of peaks, previous data referred by the reviewer is an ENCODE project (*Nature, 2014, 515:355*). As demonstrated by the quality control metrics (Supplementary Figure 1), our ChIP-seq data is of high quality, with sufficient enrichment, coverage and specificity, in addition to an optimal length of input chromatin DNA (~150bp). The stringent nature of our ChIP-seq procedure likely resulted in a loss of weak peaks, while maintaining specific loci, as such, less peaks generated than that of the ENCODE. We totally agree with the reviewers that *“it is important to clarify what promotes binding of myogenin”*, and highly appreciate the reviewer’s intriguing hypothesis: *“RXR primes within a short time the sites that will be later occupied by Myogenin”* or *“Myod promotes Myogenin binding”*. We are very interested in developing multiplex of biochemical, molecular and genetic approaches to address these important issues in a distinct scope of future studies. In the current study, we focus on the redistribution of myogenin in chromatin and the epigenetic dynamics coupled to rexinoid responsive expression. We thank the reviewer sincerely for these important questions.

Minor comment:

We have now described the *“statistical tool used”* in the Methods section.

Reviewer #2:

The reviewer asked that *“Does the Bex treatment accelerate the muscle differentiation or is it forcing some molecular mechanisms that normally are repressed?”* In line with *“Supplementary*

Figure 3 suggests that RXR activation determines an acceleration of myogenesis”, we have reported previously that bexarotene promotes myoblast differentiation through promoting exit from the cell cycle as well as a direct regulation of MyoD expression (*Nucleic Acids Res.* 2017, 45:11236). Regarding “authors should also analyse the MT4 time point in C2C12 and to knock down the RXR receptor”, we have found previously that shRNA knockdown of RXR α attenuates the positive effect of bexarotene on the differentiation and fusion of myoblasts, but knockdown of RXR β does not (*J Biol Chem.* 2016, 291:3090 and unpublished results). While RXR α is the most abundant isoform, RXR γ is minimum in both primary and C2C12 myoblasts (*J Biol Chem.* 2016, 291:3090). We thank the reviewer for the insight.

Major comments:

- 1) The reviewer commented that “*This result should be confirmed with in vitro data. One possibility is to transfect constructs containing the 2 different consensus and to perform a ChIP of Myogenin with or without Bex and RXR depletion.*” Indeed, this approach has been tested previously. While transfected plasmid DNA can associate with histones to form chromatin like structure. the organization does not mimic endogenous nucleosome, as the histone octamers are randomly positioned on plasmid DNA in a fluid arrangement lacking H1 (*J Biol Chem.* 2008, 283:4595; *Genome res.*, 2006, 16:1517; *Biotechnology & Biotechnological Equipment*, 2009, 23, 1044). In addition, this approach could not take into account other influencing factors including nuclear compartmentalization, chromatin architecture, nucleosome remodeling and histone modifications.
- 2) The reviewer commented that “*the authors found 45% of consensus for Myogenin in C2C12 differentiated 24h while in the MyoG ChIP presented in the work they found 21%*”. Sorry for the confusion, the 21% is for the peaks that are unique to control. For total population of the peaks in control condition, is 47.5% of consensus, which is comparable with the 45% for the ENCODE data. The reviewer also questioned that “*Why they have only 50% of published binding sites? Is it possible that the quality of differentiation is poor? Maybe some quality controls on differentiation could help in the evaluation.*” Indeed, we have performed quality controls (Supplementary Figure 1), which shows that our ChIP-seq is of high quality, with sufficient enrichment, coverage and specificity. There are over 14-million E-box motifs found across the mouse genome, while 2000-3000 genes are up-regulated in early myoblast differentiation. To minimize noise level and select specific binding sites, we used a high stringency ChIP protocol, which resulted in lesser number of peaks. If the same pipeline and parameter applied, the length of chromatin DNA is 150-bp for our data and 90-bp for the ENCODE. Fewer peaks (13,788) are called with the ENCODE data with our parameter than that in the ENCODE file (24,360). Still we used the peak file provided by the ENCODE, as other relevant outputs are similar. For example, the number of peaks per gene is 1.9 and 2.0 for the peaks called with our parameter and provided by the ENCODE, respectively. When normalized for peak numbers, the number of E-boxes per peak in our data is nearly identical to the ENCODE. Regarding the quality of differentiation, for each chromatin isolation, a portion of cells from the same plating are routinely subjected to differentiation analysis to ensure the quality of cells to be used for the ChIP procedure.
- 3) The reviewer stated that “*The authors should try to perform the Bex-treatment on primary myoblasts to prove the reliability of the treatment and the translational potential.*” Indeed, we have established previously the positive effect of bexarotene on

the differentiation and fusion of primary myoblasts and the ability of bexarotene to augment MyoD and myogenin gene expression in early primary myoblast differentiation (*J Biol Chem.* 2016, 291:3090).

- 4) The reviewer commented that “*in primary myoblasts I can see only one additional consensus nucleotide*”. Sorry that the image is too small to discern the two additional nucleotides. We have now enlarged the image to make the detail visible.
- 5) The reviewer stated that “*they should shown a zoom in of the region containing the peak*”. This request has been met, a zoom in of the regions containing the myogenin peaks has been provided.
- 6) The reviewer commented that “*I could see an increase of the recruitment at promoters (not enhancers).*” The sentence has now been amended to “Taken together, p300 is differentially recruited to promoters and enhancers in that p300 may mediate RXR signaling through distinct functional modes.”
- 7) The reviewer commented that “*Western blots with total P300 and histone marks levels can elucidate if bex-treatment increase the total amount of protein/histone or determine their redistribution on the genome*”. We have now added western blots for global level p300 and histone marks as requested (Figure 4a and b).
- 8) The reviewer recommended “*to look another histone modification (H3K9me3 or another repressive modification) at least to show on the same track a different trend*”. We thank the reviewer for the recommendation and have now deposited our H3K27me3 ChIP-seq data in GEO and included the data in Fig. 3d, 3e, 5a, 5c and 6b-d. The H3K27me3 ChIP-seq was performed in parallel with p300 and histone acetylation as a repressor mark control.
- 9) The reviewer stated that “*Authors should analyse a well-described enhancer (not putative) to reinforce their speculations*”. In fact, studies have characterized the Mybph loci as a myogenic super enhancer (dbSUPER database, *Nuclei Acids Res.* 2016, 44:D16; https://ruor.uottawa.ca/bitstream/10393/40032/5/Abdelkarim_Basma_2020_thesis.pdf). *In vitro* assays have also been used to establish its enhancer activity in myoblasts and myocytes (<https://thesis.library.caltech.edu/11058/7/DeSalvo-Thesis-2018.pdf>).
- 10) The reviewer suggested that “*A model to summarize all results can help the reader*”. This request has been met, a model to summarize the results has been presented in the revised manuscript. We thank the reviewer for the suggestion.

Minor comments:

- 1) We have now formatted correctly the reference in question and thank the review for the diligence.
- 2) As requested, we have now moved previous Figure 1a and b to supplementary Figure 1.
- 3) We have now provided a track of myogenin in Figure 1 to “*help the reader to evaluate the ChIP-seq*”.
- 4) We have now added appropriate legend for previous Figure 4a (currently Figure 4C).
- 5) We have now provided a color legend for Figure 6d.

We thank you again for your time and consideration,

Qiao Li MD, PhD
Associate Professor
Department of Cellular and Molecular Medicine
Faculty of Medicine, University of Ottawa
451 Smyth Road, Room 2537
Ottawa, Ontario, Canada K1H 8M5

Tel: +1 613 562 5800 ext. 8491 (Office) / 8488 (Lab)

Webpage: <http://www.med.uottawa.ca/patho/eng/li.html>
<http://med.uottawa.ca/cellular-molecular/people/li-qiao>

Reviewers' comments:

Reviewer #1 (Remarks to the Author):

The revised version of the manuscript by Khilji and colleagues addresses some of the comments raised following the initial submission but does not provide insights regarding the potential molecular mechanism(s) accounting for distinct binding selectivity of Myogenin following BEX treatment. For example, is the differential binding selectivity caused by the increased protein levels of Myogenin? Based on the ChIP signal intensity in Figure 1C, it appears that Myogenin may be present at lower levels at the BEX unique sites. Manipulation of Myog levels will help to clarify this aspect. Since the authors indicate that BEX induces Myod, which in turn regulates Myogenin levels, it is hard to conclude without further analyses that "RXR signaling modifies the chromatin state distribution of myogenin, promoting the binding preference of myogenin for poised enhancers and a distinct motif", as stated in the Abstract and Discussion.

Reviewer #2

In the revised version of this manuscript authors performed additional experiments, improving the quality of the work. I am satisfied of how some of them address my concerns, however I have still some few comments on the new data.

1) The reviewer commented that "This result should be confirmed with in vitro data. One possibility is to transfect constructs containing the 2 different consensus and to perform a ChIP of Myogenin with or without Bex and RXR depletion." Indeed, this approach has been tested previously. While transfected plasmid DNA can associate with histones to form chromatin like structure. the organization does not mimic endogenous nucleosome, as the histone octamers are randomly positioned on plasmid DNA in a fluid arrangement lacking H1 (J Biol Chem. 2008, 283:4505; Genome res, 2006, 16:1517; Biotechnology & Biotechnological Equipment, 2009, 23, 1044). In addition, this approach could not take into account other influencing factors including nuclear compartmentalization, chromatin architecture, nucleosome remodeling and histone modifications.

The use of the in vitro assay to confirm the binding to the DNA is widely used by the scientific community (PNAS April 17, 2018 115 (16) E3692-E3701; Briefings in Functional Genomics, Volume 16, Issue 3, May 2017, Pages 171–180; Mol Cell Biol. 2003 Jun; 23(11): 3837–3846). One of the 3 papers cited from the authors in their rebuttal (Biotechnology & Biotechnological Equipment, 2009) shows that a transfected plasmid can reproduce the chromatin environment as stated in the conclusion: "After transfection in mammalian cells, plasmid DNA enters the nucleus where it is complexed with histones to acquire nucleoprotein structure similar to that of the native chromatin. Under this form its transcription, recombination and repair are carried out by the same cellular mechanisms that carry out chromosomal transcription, recombination and repair." This experiment can improve the quality of the work, because can demonstrate uniquely the effect of Bex on MyoG binding. On the other hand, this experiment can also reveal that the chromatin environment is more important than the DNA sequence itself adding an important insight to the work.

8) The reviewer recommended "to look another histone modification (H3K9me3 or another repressive modification) at least to show on the same track a different trend". We thank the reviewer for the recommendation and have now deposited our H3K27me3 ChIP-seq data in GEO and included the data in Fig. 3d, 3e, 5a, 5c and 6b-d. The H3K27me3 ChIPseq was performed in parallel with p300 and

histone acetylation as a repressor mark control.

I did not find the quality controls for the H3K27me3 ChIP-seq. Genomic regions analyzed in the paper, being transcriptionally active, are not representative of H3K27me3 ChIP-seq. H3K27me3 distribution at TSS is strictly requested to show that the ChIP-seq worked correctly.

QIAO LI MD, PhD

613 562 5800 ext. 8491
Qiao.Li@uOttawa.ca

April 14, 2020

Dear Reviewers,

We thank you very much for your critique and valuable suggestions. We have now provided requested data and addressed issues raised by you. Specific response are as follows:

Reviewer #1:

The reviewer asked that “*is the differential binding selectivity caused by the increased protein levels of Myogenin? Based on the ChIP signal intensity in Figure 1C, it appears that Myogenin may be present at lower levels at the BEX unique sites*”. The differential binding is caused likely by contributing factors including E-box components, density and flanking sequences. While the protein level of myogenin is higher in bexarotene treated myoblasts than in control, ChIP signals are comparable at sites shared between treated and control myoblasts, but lower at sites unique to control and higher at unique to bexarotene in treated myoblasts compared to control. We have now toned down the conclusion and stated that further studies are important for understanding other factors of myogenic networks, in response to the comment: “*Since the authors indicate that BEX induces Myod, which in turn regulates Myogenin levels, it is hard to conclude without further analyses that "RXR signaling modifies the chromatin state distribution of myogenin, promoting the binding preference of myogenin for poised enhancers and a distinct motif", as stated in the Abstract and Discussion*”.

Reviewer #2:

1. The reviewer commented “*The use of the in vitro assay to confirm the binding to the DNA is widely used by the scientific community (PNAS April 17, 2018 115 (16) E3692-E3701; Briefings in Functional Genomics, Volume 16, Issue 3, May 2017, Pages 171-180; Mol Cell Biol. 2003 Jun; 23(11): 3837-3846).*” Regarding the three papers cited by the reviewer: a) Rastogi et al. (PNAS. 2018, 115: E3692) introduce a sequence to affinity computational modeling named as No Read Left Behind (NRLB) to maximize information from a single round of selection in SELEX experiments. The authors suggest that NRLB predictions of protein binding sites are accurate enough that further validation of TF-DNA affinity by *in vitro* assays, such as EMSAs, may no longer be required. Nevertheless, the authors did not use or discuss ChIP assays on transient transfection of plasmid DNA in this study. b) Orenstein and Shamir (Briefings in Functional Genomics. 2017, 16: 171) review different high throughput methodologies for measuring protein–DNA binding, including ChIP-ChIP, ChIP-seq, universal protein-binding microarrays (uPBMs), and High-throughput SELEX (HT-SELEX). The authors discuss advantages and limitations of different technologies and associated computational approaches for data analyses, but no discussion on the use of transient transfection of plasmid DNA in combination with ChIP to quantify transcription factor binding. c) Zhao et al. (Mol Cell Biol. 2003, 23: 3837) tested transcriptional activation of a reporter by transiently transfecting Early B-cell factor (EBF) in multiple cell types followed by luciferase reporter assays. Electrophoretic mobility shift assays were also used to analyze

the DNA binding of EBF. However, the authors did not use the approach of plasmid transfection in combination with ChIP in this study.

The reviewer stated that “(*Biotechnology & Biotechnological Equipment, 2009*) shows that a transfected plasmid can reproduce the chromatin environment as stated in the conclusion: ???After transfection in mammalian cells, plasmid DNA enters the nucleus where it is complexed with histones to acquire nucleoprotein structure similar to that of the native chromatin. Under this form its transcription, recombination and repair are carried out by the same cellular mechanisms that carry out chromosomal transcription, recombination and repair.???” Indeed, as refereed by the reviewer, the authors made the statement. However, the actual experimental data showed that the digest patterns of the transfected plasmid DNA exhibiting an uneven nucleosomal ladder that differs from that observed for the native chromatin. As such the authors concluded that while transfected plasmid DNA acquires nucleoprotein structure, similar to the native chromatin, there are differences for reason unclear to them. In fact, their observation has been reported by previous studies. Jeong and Stein (*Nucleic Acids Res. 1994, 22: 370*) reported that while nucleosome like patterns were formed on transfected plasmid DNA, nucleosome ladders were anomalous and lacked the multiple repeat unit (180-190 bp) seen for the native chromatin. Using DNA plasmids of varying size in multiple cell types, the authors found that only 10% of transfected plasmid generated a typical nucleosome ladder. Likewise, Hebbar and Archer (*J Biol Chem. 2008, 283: 4595*) found that H1 is underrepresented on transfected plasmid, an indicative of irregular nucleosomal organization, which may underlie a different mechanism of activation of the transient template.

The reviewer also commented that “*This experiment can improve the quality of the work, because can demonstrate uniquely the effect of Bex on MyoG binding. On the other hand, this experiment can also reveal that the chromatin environment is more important than the DNA sequence itself adding an important insight to the work*”. We indeed appreciate these points of considerations. Trained in classic chromatin research, I am passionate about these fundamental issues and have in the past employed the transfected plasmid approach. Depending cellular context, flanking sequences, or the topology of DNA regions of interests, caveats out-weight the advantages of the approach. Given today’s technology, we are actively developing a strategy of CRISPR-mediated genome editing in an attempt to address these issues *in vivo*. We thank the reviewer sincerely for these important suggestions.

2. The reviewer stated that “*I did not find the quality controls for the H3K27me3 ChIP-seq. Genomic regions analyzed in the paper*”. “*H3K27me3 distribution at TSS is strictly requested to show that the ChIP-seq worked correctly*”. We have now provided the quality controls and the H3K27me3 distribution across TSS (Figure S4). We thank the reviewer for the insight.

We thank you again for your time and consideration,

Qiao Li MD, PhD
Associate Professor
Department of Cellular and Molecular Medicine
Faculty of Medicine, University of Ottawa, Ottawa, ON, Canada K1H 8M5