# Supplementary material for: Dissecting myogenin-mediated retinoid X receptor signaling in myogenic differentiation
Source: Commun Biol. 2020 Jun 18;3:315. doi: 10.1038/s42003-020-1043-9 (PMC7303199; doi:10.1038/s42003-020-1043-9)
Supplement: Supplementary file 1 — Supplementary Information [file 42003_2020_1043_MOESM1_ESM.pdf]

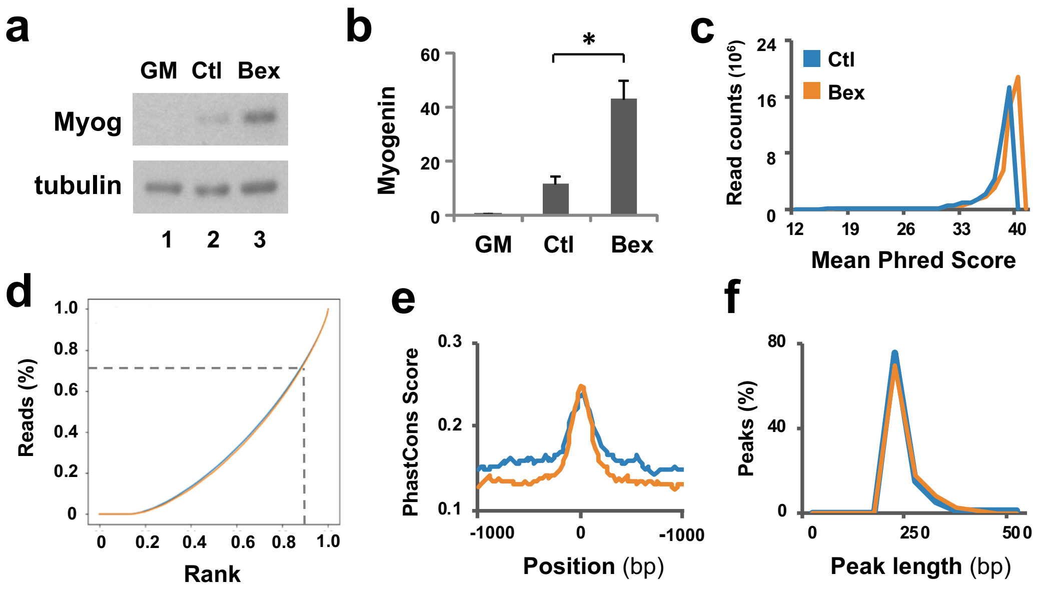

Supplementary Figure 1. Quality control of myogenin ChIP-seq.
(a) C2C12 myoblasts were differentiated for 24-hour in the absence (Ctl) or presence of bexarotene (Bex), followed by Western blotting of myogenin protein levels. Proliferating myoblasts (GM) were included with β-tubulin as a loading control. (b) Quantification of myogenin protein levels as a fold change relative to proliferating myoblasts (error bars: SD; n = 4; *, $p \leq 0.05$, Student's t-test). (c) Base quality scores for myogenin sequencing data. (d) ChIP enrichment plot shows the percentage of reads in a given percent of mappable genome, where rightward deflection indicates the extent of ChIP signal enrichment. (e) The average PhastCons conservation scores of myogenin peaks. (f) Distribution of the length of myogenin peaks.

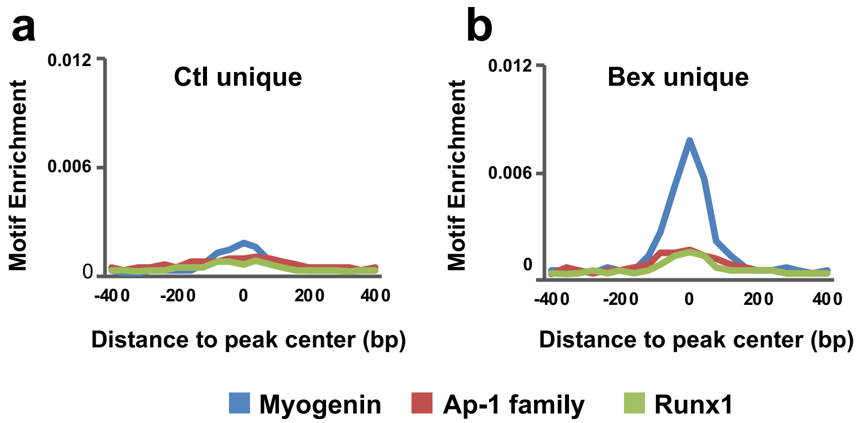

Supplementary Figure 2. Motif enrichment at myogenin loci.
(a) Motif enrichment at myogenin loci unique to C2C12 myoblasts differentiated for 24-hour in the absence or (b) presence of bexarotene (Ctl or Bex).

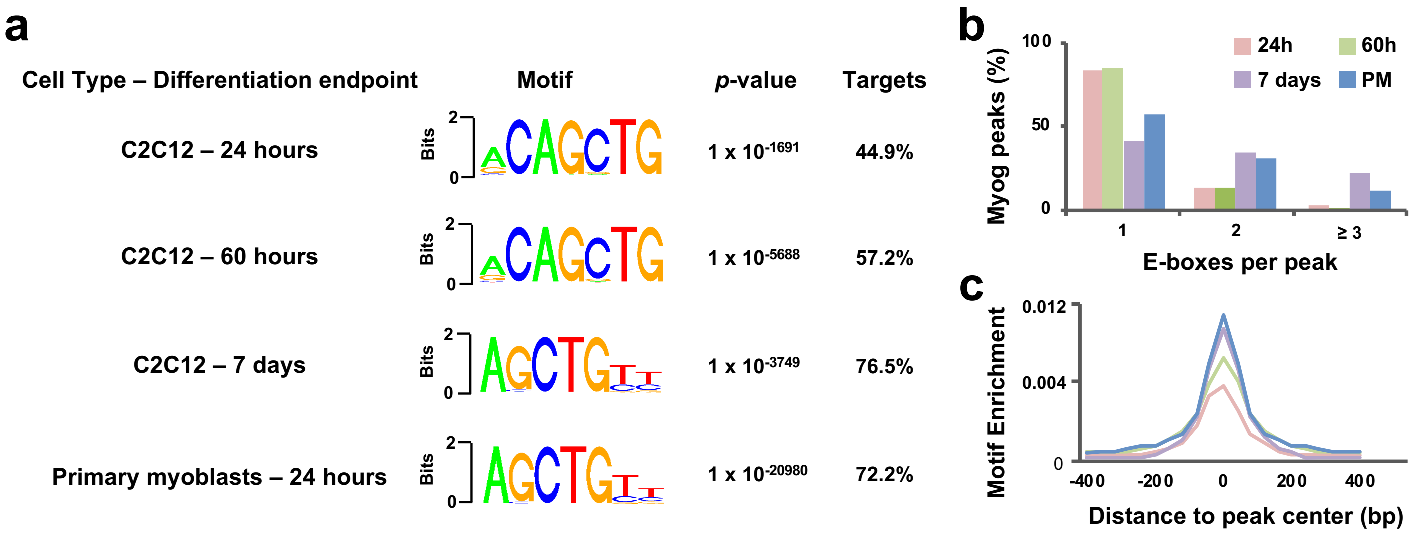

Supplementary Figure 3. Motif analysis of publicly available myogenin ChIP-seq. (a) Myogenin loci in myoblasts differentiated for 24-, 60-hour, and 7 days as well as primary myoblasts (PM) differentiated for 24-hour were subjected to *de novo* motif analysis. The most significant motif in each condition, its associated *p*-value and the percentage of target loci harboring the motif are shown. (b) Bar graph displays the percentage of myogenin peaks associated to a specified number of E-box per peak. (c) Enrichment of motifs shown across myogenin loci.

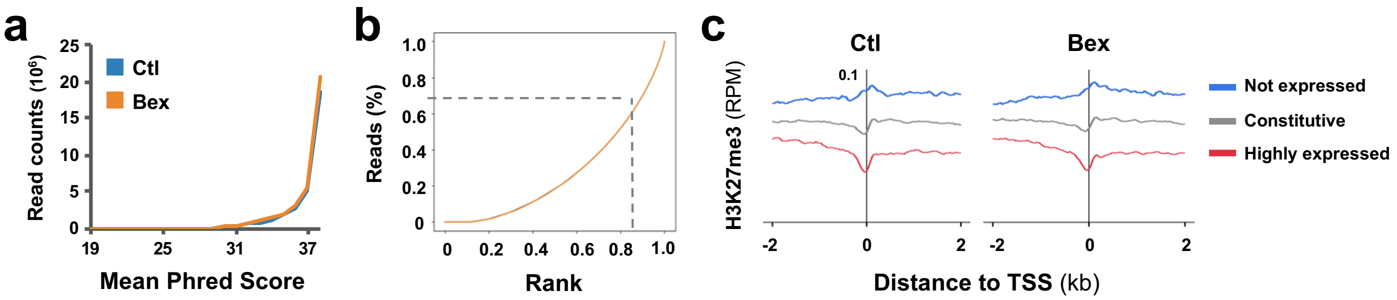

Supplementary Figure 4. Quality control of H3K27me3 ChIP-seq.
(a) C2C12 myoblasts were differentiated for 24-hour in the absence (Ctl) or presence of bexarotene (Bex) and subjected to H3K27me3 ChIP-seq. Quality score distribution shows majority of reads were attributed with a Phred score above 30, equating to 99.9% base call accuracy. (b) ChIP enrichment plot presents the percentage of reads in a given percent of mappable genome. Rightward deflection of the curve indicates ChIP enrichment. (c) The total population of ENSEMBL genes from RNA-seq expression profiling of proliferating myoblasts and myoblasts differentiated for 24-hour were categorized into three groups (GSE94560). The average enrichment of H3K27me3 (reads per million) with respect to the TSS of each gene group is shown. H3K27me3 was prominently enriched around the TSS of inactive genes, consistent with its role in gene silencing.
