# Supplementary material for: Dissecting myogenin-mediated retinoid X receptor signaling in myogenic differentiation
Source: Commun Biol. 2020 Jun 18;3:315. doi: 10.1038/s42003-020-1043-9 (PMC7303199; doi:10.1038/s42003-020-1043-9)
Supplement: Supplementary file 2 — Description of Additional Supplementary Files [file 42003_2020_1043_MOESM2_ESM.pdf]

Description of Additional Supplementary Files

File Name: **Supplementary Data 1**
Description: Source file for Figure 1-6
