# Supplementary material for: Dissecting myogenin-mediated retinoid X receptor signaling in myogenic differentiation
Source: Commun Biol. 2020 Jun 18;3:315. doi: 10.1038/s42003-020-1043-9 (PMC7303199; doi:10.1038/s42003-020-1043-9)
Supplement: Supplementary file 4 — Reporting Summary [file 42003_2020_1043_MOESM4_ESM.pdf]

natureresearch

Corresponding author(s): Qiao Li

Last updated by author(s): May 4, 2020

Reporting Summary

Nature Research wishes to improve the reproducibility of the work that we publish. This form provides structure for consistency and transparency in reporting. For further information on Nature Research policies, see Authors & Referees and the Editorial Policy Checklist.

Statistics

For all statistical analyses, confirm that the following items are present in the figure legend, table legend, main text, or Methods section.

n/a	Confirmed	
☐	☒	The exact sample size (n) for each experimental group/condition, given as a discrete number and unit of measurement
☐	☒	A statement on whether measurements were taken from distinct samples or whether the same sample was measured repeatedly
☐	☒	The statistical test(s) used AND whether they are one- or two-sided<br>*Only common tests should be described solely by name; describe more complex techniques in the Methods section.*
☐	☒	A description of all covariates tested
☐	☒	A description of any assumptions or corrections, such as tests of normality and adjustment for multiple comparisons
☐	☒	A full description of the statistical parameters including central tendency (e.g. means) or other basic estimates (e.g. regression coefficient) AND variation (e.g. standard deviation) or associated estimates of uncertainty (e.g. confidence intervals)
☐	☒	For null hypothesis testing, the test statistic (e.g. F, t, r) with confidence intervals, effect sizes, degrees of freedom and P value noted<br>*Give P values as exact values whenever suitable.*
☒	☐	For Bayesian analysis, information on the choice of priors and Markov chain Monte Carlo settings
☒	☐	For hierarchical and complex designs, identification of the appropriate level for tests and full reporting of outcomes
☒	☐	Estimates of effect sizes (e.g. Cohen's d, Pearson's r), indicating how they were calculated

Our web collection on statistics for biologists contains articles on many of the points above.

Software and code

Policy information about availability of computer code

Data collection	Illumina HiSeq sequencing and associated software was used for data collection.
Data analysis	The software used to analyze data in this study are publicly available and are described in the methods section.

For manuscripts utilizing custom algorithms or software that are central to the research but not yet described in published literature, software must be made available to editors/reviewers. We strongly encourage code deposition in a community repository (e.g. GitHub). See the Nature Research guidelines for submitting code & software for further information.

Data

Policy information about availability of data

All manuscripts must include a data availability statement. This statement should provide the following information, where applicable:

- Accession codes, unique identifiers, or web links for publicly available datasets
- A list of figures that have associated raw data
- A description of any restrictions on data availability

All ChIP-seq datasets have been deposited in the NCBI Gene Expression Omnibus (GEO) under accession number GSE139942. The source data for the main figures are provided in the Source Data file. All other relevant data supporting the key findings of this study are available within the article and its Supplementary Information or from the corresponding author upon request.

Field-specific reporting

Please select the one below that is the best fit for your research. If you are not sure, read the appropriate sections before making your selection.

☒ Life sciences ☐ Behavioural & social sciences ☐ Ecological, evolutionary & environmental sciences

For a reference copy of the document with all sections, see nature.com/documents/nr-reporting-summary-flat.pdf

Life sciences study design

All studies must disclose on these points even when the disclosure is negative.

Sample size	N/A
Data exclusions	No data were excluded.
Replication	Two biological replicates for RNA-seq data were used in the analysis.<br>For qPCR, qChIP, and western analysis a minimum of 3 replicates were used to confirm the observed trend.
Randomization	We utilized an in vitro cell model. Cells were grown and split into plates for control and treatment groups randomly.
Blinding	Not relevant for this study.

Reporting for specific materials, systems and methods

We require information from authors about some types of materials, experimental systems and methods used in many studies. Here, indicate whether each material, system or method listed is relevant to your study. If you are not sure if a list item applies to your research, read the appropriate section before selecting a response.

Materials & experimental systems

n/a	Involved in the study	
☐	☒	Antibodies
☐	☒	Eukaryotic cell lines
☒	☐	Palaeontology
☒	☐	Animals and other organisms
☒	☐	Human research participants
☒	☐	Clinical data

Methods

n/a	Involved in the study	
☐	☒	ChIP-seq
☒	☐	Flow cytometry
☒	☐	MRI-based neuroimaging

Antibodies

Antibodies used	All antibodies used in this study are commercially available.<br><br>ChIP-seq: Myogenin (sc-12732); p300 (sc-584); H3K27me3 (ab6002)<br>Western: hybridoma F5D for myogenin and hybridoma E7 for β-tubulin
Validation	Myogenin (sc-12732): https://www.scbt.com/p/myogenin-antibody-f5d<br>p300 (sc-584): https://www.scbt.com/p/p300-antibody-n-15<br>H3K27me3 (ab6002): https://www.abcam.com/histone-h3-tri-methyl-k27-antibody-mabcam-6002-chip-grade-ab6002.html<br><br>Myogenin hybridoma F5D: https://dshb.biology.uiowa.edu/F5D<br>β-tubulin hybridoma E7: https://dshb.biology.uiowa.edu/E7_2

Eukaryotic cell lines

Policy information about cell lines

Cell line source(s)	C2C12 cells: American Type Culture Collection (ATCC)
Authentication	Authentication by ATCC.
Mycoplasma contamination	Cells were not tested for mycoplasma contamination.
Commonly misidentified lines<br>(See ICLAC register)	N/A

ChIP-seq

Data deposition

☒ Confirm that both raw and final processed data have been deposited in a public database such as GEO.

☒ Confirm that you have deposited or provided access to graph files (e.g. BED files) for the called peaks.

Data access links<br>*May remain private before publication.*	https://www.ncbi.nlm.nih.gov/geo/query/acc.cgi?acc=GSE139942
Files in database submission	GSM4150361 C2C12_Input_p300_Histones<br>GSM4150362 C2C12_p300_Ctl<br>GSM4150363 C2C12_p300_Bex<br>GSM4367470 H3K27me3_Ctl<br>GSM4367471 H3K27me3_Bex<br><br>GSM4150364 C2C12_Input_Myog<br>GSM4150365 C2C12_Myog_Ctl<br>GSM4150366 C2C12_Myog_Bex
Genome browser session<br>(e.g. UCSC)	NA

Methodology

Replicates	Western: 3 biological replicates<br>qChIP and qPCR: 3 technical replicates
Sequencing depth	All datasets are single-end 50bp reads with an average of 27.1 million uniquely mapped reads.
Antibodies	Myogenin (sc-12732), p300 (sc-584), and H3K27me3 (ab6002)
Peak calling parameters	HOMER findPeaks was used for peak calling with the following parameters: -style factor -region.
Data quality	Data quality control was perfomed with FastQC, Qualimap and deepTools to ensure sufficient ChIP enrichment and coverage.
Software	Software used in this study includes:<br>SAMtools<br>BWA-MEM<br>BEDTools<br>ngsplot<br>MEME Suite<br>deepTools<br>HOMER<br>IGV<br>R Studio
